# Which type of feedback—Positive or negative- reinforces decision recall? An EEG study

**DOI:** 10.3389/fnsys.2024.1524475

**Published:** 2025-01-08

**Authors:** Michela Balconi, Laura Angioletti, Roberta A. Allegretta

**Affiliations:** ^1^International research center for Cognitive Applied Neuroscience (IrcCAN), Università Cattolica del Sacro Cuore, Milan, Italy; ^2^Research Unit in Affective and Social Neuroscience, Department of Psychology, Università Cattolica del Sacro Cuore, Milan, Italy

**Keywords:** feedback valence, recall, decision-making, EEG, theta, alpha, beta, gamma

## Abstract

This study examines the impact of positive and negative feedback on recall of past decisions, focusing on behavioral performance and electrophysiological (EEG) responses. Participants completed a decision-making task involving 10 real-life scenarios, each followed by immediate positive or negative feedback. In a recall phase, participants’ accuracy (ACC), errors (ERRs), and response times (RTs) were recorded alongside EEG data to analyze brain activity patterns related to recall. Results indicate that accurately recalled decisions with positive feedback had slower RTs, suggesting an attentional bias toward positive information that could increase cognitive load during memory retrieval. A lack of difference in recall accuracy implies that social stimuli and situational goals may influence the positivity bias. EEG data showed distinct patterns: lower alpha band activity in frontal regions (AF7, AF8) for both correct and incorrect decisions recall, reflecting focused attention and cognitive control. Correctly recalled decisions with negative feedback showed higher delta activity, often linked to aversive processing, while incorrect recalls with negative feedback showed higher beta and gamma activity. A theta band feedback-dependent modulation in electrode activity showed higher values for decisions with negative feedback, suggesting memory suppression. These findings suggest that recalling decisions linked to self-threatening feedback may require greater cognitive effort, as seen in increased beta and gamma activity, which may indicate motivational processing and selective memory suppression. This study provides insights into the neural mechanisms of feedback-based memory recall, showing how feedback valence affects not only behavioral outcomes but also the cognitive and emotional processes involved in decision recall.

## 1 Introduction

To gain a clearer understanding of the outcomes of a decision and to improve future decision-making, people need feedback from external sources. Behaviors that lead to negative outcomes, such as punishment or negative feedback, are less likely to be repeated, while those that produce positive results, like rewards or positive feedback, tend to encourage the behavior to occur more often (Kobza et al., 2012).

The fundamental role of feedback in shaping behavior is clear and well-documented by several research (Mangiapanello and Hemmes, 2015; Crivelli et al., 2023; Balconi et al., 2024b), yet its influence extends beyond immediate decision-making, affecting how individuals remember and learn from their experiences. Feedback, whether positive or negative, not only guides future actions but also plays a pivotal role in how well these experiences are encoded and later recalled (Hattie and Timperley, 2007). Notably, research indicates that the valence of feedback significantly influences memory recall: a study by Höltje and Mecklinger (2020) demonstrated that positive feedback improves memory encoding, resulting in better recall compared to negative feedback. This is consistent with findings from Albrecht et al. (2023), who observed that immediate positive feedback can affect recall, implying a complex interaction between feedback valence and memory processes.

Studies conducted on healthy individuals suggest that they frequently exhibit a positivity bias, tending to remember events as more favorable than they were (Schacter et al., 2011; Levine et al., 2012). This bias likely stems from an adaptive mechanism aimed at preserving self-esteem if perceived as threatened. Indeed, because self-threatening information tends to be distressing, people make considerable efforts to eliminate such memories (Anderson and Green, 2001; Gagnepain et al., 2014). The mnemic neglect effect (MNE) further explains (Sedikides and Green, 2005, 2006) this selective forgetting suggesting that individuals are driven to protect or enhance the positivity of their self-concept and tend to disproportionately overlook the processing of negative, self-threatening feedback. As a result, self-threatening feedback is processed superficially, resulting in weaker recall due to shallow processing.

Nonetheless, some research also showed that human cognition and behavior are influenced by a negativity bias, where people tend to prioritize negative information over positive information across different psychological contexts (Norris et al., 2019). This bias is believed to have evolved as a survival mechanism, with avoiding harmful stimuli being more essential to survival than seeking out beneficial ones (Norris et al., 2019).

To better understand how different feedback valence affects the recall of a decision, important support comes from neuroscience through the use of electroencephalography (EEG). Indeed, brain activity related to specific perceptual, cognitive, and emotional processes can be examined through the analysis of EEG frequency bands (delta, theta, alpha, beta, and gamma) and the interpretation of their functional significance providing deeper insights into the neural mechanisms underlying these processes. Notably, low-frequency bands (delta and theta) generally exhibit an increase during emotional processes (Balconi et al., 2015, 2019), while high-frequency bands (alpha, beta and gamma) are typically associated with cognitive effort, processing, engagement, and mechanisms of focused attention (Wróbel, 2000; Pizzagalli, 2007; Engel and Fries, 2010).

To the best of our knowledge, there are limited researches focusing specifically on the recall of decisions influenced by varying feedback valence in healthy populations. Therefore, our study combined behavioral measures – response accuracy, errors and response times (RTs) – with electrophysiological data to investigate how decision-makers recalled previously made decisions under different feedback valences. In the first part of the task, participants were presented with 10 real-life decision-making scenarios where they could choose between two alternatives. Regardless of their choice, all participants received either immediate negative feedback for five scenarios and immediate positive feedback for the other five. In the second part of the task, participants were asked to recall their responses for each scenario.

Based on previous considerations, firstly we explored potential differences in response accuracy and errors for the recall of decisions based on feedback valence. We expected to find that participants would accurately recall decisions associated with positive feedback more than those linked to negative feedback. Research suggests that healthy individuals often exhibit a positivity bias, remembering events more favorably than they were (Schacter et al., 2011; Levine et al., 2012), which serves to preserve self-esteem. This can be explained by the MNE (Sedikides and Green, 2005, 2006), which suggests that individuals tend to process self-threatening feedback superficially, resulting in poorer recall of negative information. We also investigated differences in RTs in recalling decisions based on feedback valence, since they serve as indicators of information processing, cognitive effort, and decision workload (Balconi et al., 2024a). We expected longer RTs for accurately recalled decisions with positive feedback compared to those with negative feedback. Indeed, while positive stimuli are typically easier to remember, attentional biases toward them may slow cognitive processing and memory retrieval, resulting in slower RTs (Thoern et al., 2016).

Secondly, we expected a different pattern of activity for EEG frequency bands for both accurately and incorrectly recalled decisions with positive and negative feedback. Specifically, we anticipated a crucial role of high-frequency bands: we expected a general decrease of alpha band mainly in frontal areas in the recall of decisions despite feedback valence. Indeed, alpha oscillations act as gatekeepers in neural processes, influencing how attention is directed and how stored information is accessed during tasks (Knyazev, 2013). Particularly, higher alpha activity in certain areas of the brain may indicate that region is not essential for processing information, thus its activity is suppressed while other brain regions play a more significant role in the process (Klimesch et al., 2007). On the other hand, we expected also gamma and beta to increase during the recall of decisions since beta oscillations are thought to support top-down control processes that support retrieving information from memory (Miller et al., 2018), whereas gamma oscillations are associated with the encoding and retrieval of specific memory traces, which help improve the accuracy of recalled information (Lundqvist et al., 2016).

## 2 Method

### 2.1 Sample

A total of 20 participants were selected for this pilot study (Mean age = 37.35, Standard Deviation age = 15.05, age range: 22–61, with 8 males). Exclusion criteria encompassed a history of psychiatric or neurological disorders, significant head injuries or strokes, undergoing therapy with psychoactive drugs that could influence cognitive abilities or decision-making. All participants had normal or corrected vision and hearing. Participation was voluntary, with no financial compensation provided. Each participant gave written informed consent prior to the study.

The study received approval from the Ethics Committee of the Department of Psychology at the Catholic University of the Sacred Heart in Milan (approval code: 125/24 – “Evaluating Decision-Making: Awareness and Metacognitive Decision-Making”; approval date: 23rd July 2024) and was carried out in accordance with the Declaration of Helsinki (2013) and GDPR regulations (EU Regulation 2016/679) and its ethical standards.

### 2.2 Procedure

After receiving an explanation of the experimental procedure and providing informed consent, participants were seated comfortably approximately 80 cm from a computer, wearing a wearable EEG device and completed the experimental task – delivered through an online platform (PsyToolkit, version 3.4.4) (Stoet, 2010, 2017) – in a quiet environment.

### 2.3 Decision recall task

**Figure 1:**
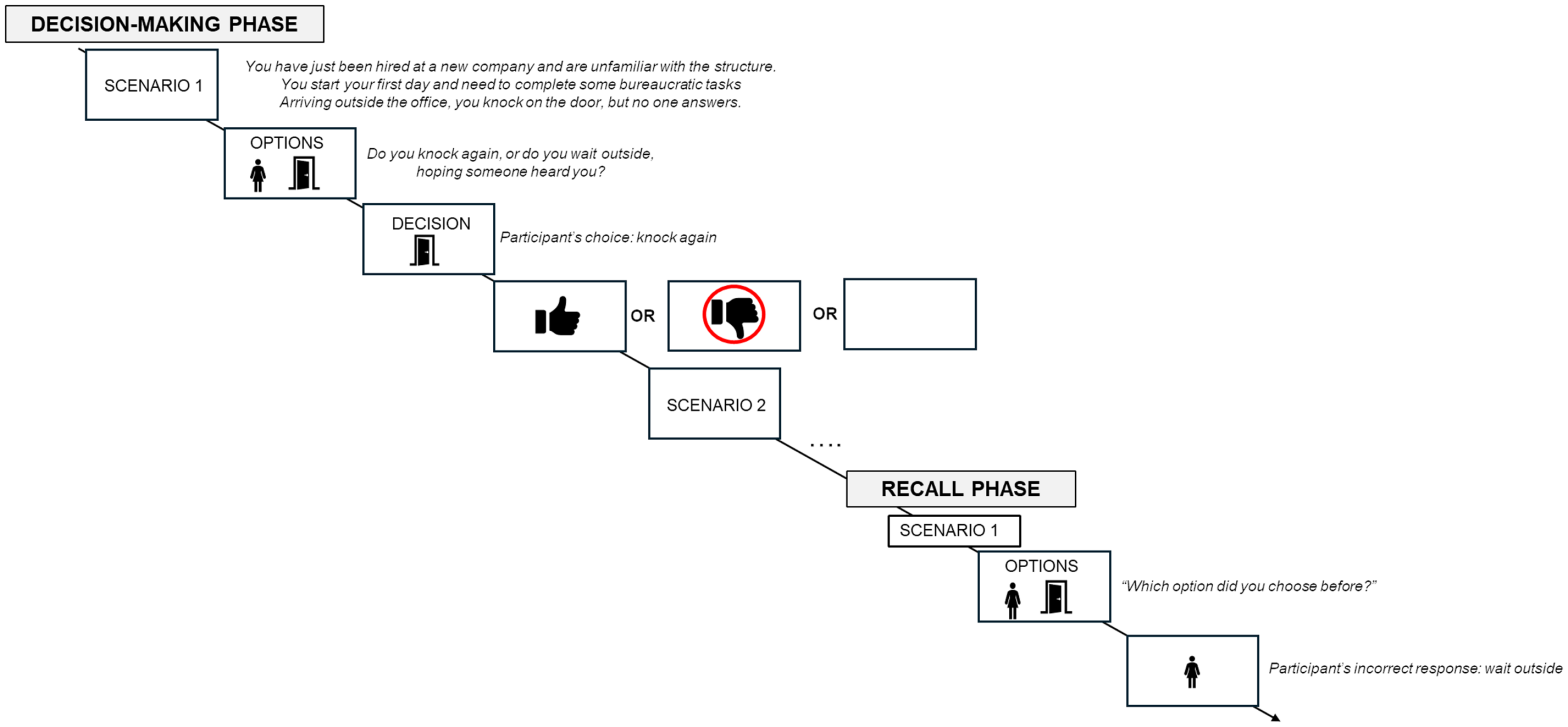
Figure represents the experimental procedure.

The experimental task was structured to examine the influence of positive and negative feedback on the recall of a previously made decision, and it was divided into two phases. In the first phase (decision-making phase), participants were shown a scenario explaining they were starting their first day at a new job and that they needed to make some decisions throughout the day (e.g., “*You have just been hired at a new company and are unfamiliar with the structure. You start your first day and need to complete some bureaucratic tasks.*”). They were then given ten scenarios and asked to choose between two options, selecting the one they believed was most appropriate. After each decision, feedback about the consequences of their choice was displayed. Regardless of the option chosen, five scenarios always provided positive feedback (e.g., “Perfect, now you know where the office is!”) and five provided negative feedback (e.g., “This decision wasted a lot of time; you should have made a different choice!”). Instead, the second phase (recall phase) aimed to assess the accuracy of participants’ recall regarding their initial decisions. Participants were presented with the same scenarios from the first phase and asked to indicate which option they chose (see Figure 1). Behavioral data, including participants’ accuracy (ACC), errors (ERRs) and response times (RTs) for each scenario were collected.

Specifically, ACC was calculated as the number of correctly recalled decisions on the total amount of decisions for both positive and negative feedback, ERRs were calculated as the number of wrongly recalled decisions on the total amount of decisions for both positive and negative feedback.

### 2.4 EEG data acquisition and processing

EEG data were collected for 120 s during both a resting state and while participants completed the recall phases of the task using a wearable, non-invasive EEG device, the Muse™ headband (version 2; InteraXon Inc., Toronto, ON, Canada). This system detects spectral activity across standard frequency bands (delta, theta, alpha, beta, and gamma) using seven dry electrodes made from conductive silver material and silicone rubber. The electrodes are arranged according to the 10–20 system (Jasper, 1958), three functions as reference points, while the remaining four are positioned on the left and right sides of the forehead: two in the frontal area (AF7 and AF8) and two in the temporoparietal area (TP9 and TP10). The data were sampled at 256 Hz, with a 50 Hz notch filter applied, and recorded via a system featuring an accelerometer, gyroscope, and pulse oximetry, all synced through the Mind Monitor mobile app via Bluetooth. Participants were instructed to minimize eye movements and blinking to reduce potential artefacts, which were later manually removed through visual inspection. These artefacts included blinks, jaw clenching, and movement. EEG data from each electrode and frequency band were converted in real time into Power Spectral Density (PSD) using Fast Fourier Transformation, applied across the delta (1–4 Hz), theta (4–8 Hz), alpha (8–13 Hz), beta (13–30 Hz), and gamma (30–44 Hz) bands. Power variations during task performance were calculated by comparing the power values during the task with baseline values.

The EEG data was recorded during the recall phase as participants recalled the decisions previously made, involving a choice between two distinct options. Following data acquisition, the EEG signal underwent an offline segmentation procedure. This process involved segmenting the signal according to the feedback associated with each decision outcome in the first phase of the task (i.e., decision-making phase), categorizing segments into those associated with positive and negative feedback and further differentiating between correct and incorrect responses. This segmentation enables a comprehensive analysis of the neural activity patterns underlying distinct decision outcomes and feedback types, facilitating a deeper understanding of the cognitive mechanisms involved in decision-making.

### 2.5 Data analysis

The assumption of normality for the distribution was preliminarily tested and confirmed through the application of kurtosis and skewness tests. Based on this evidence, four distinct repeated measure ANOVAs with Feedback (2: positive, negative) as independent within variables was applied to the ACC and RTs of correctly recalled decisions (i.e., correct responses), to ERRs (i.e., incorrect responses, which are wrongly recalled decisions) and to the RTs of the incorrect responses as dependent variables.

Regarding EEG data, four distinct repeated measure ANOVAs with Feedback (2: positive, negative) and Electrode (4: AF7, AF8, TP9, TP10) as independent within variables was applied to the variations of EEG frequency bands for segments related to correct and incorrect responses of recalled decisions as dependent variables. When required, the degrees of freedom for each ANOVA test were adjusted using the Greenhouse–Geisser epsilon. Following this, significant interactions were explored through pairwise comparisons to examine simple effects. To control potential biases due to multiple comparisons, the Bonferroni correction was applied. The effect sizes for statistically significant results were quantified using eta squared (*η*^2^), with a significance threshold set at *α* = 0.05.

## 3 Results

### 3.1 Behavioral results

**Figure 2:**
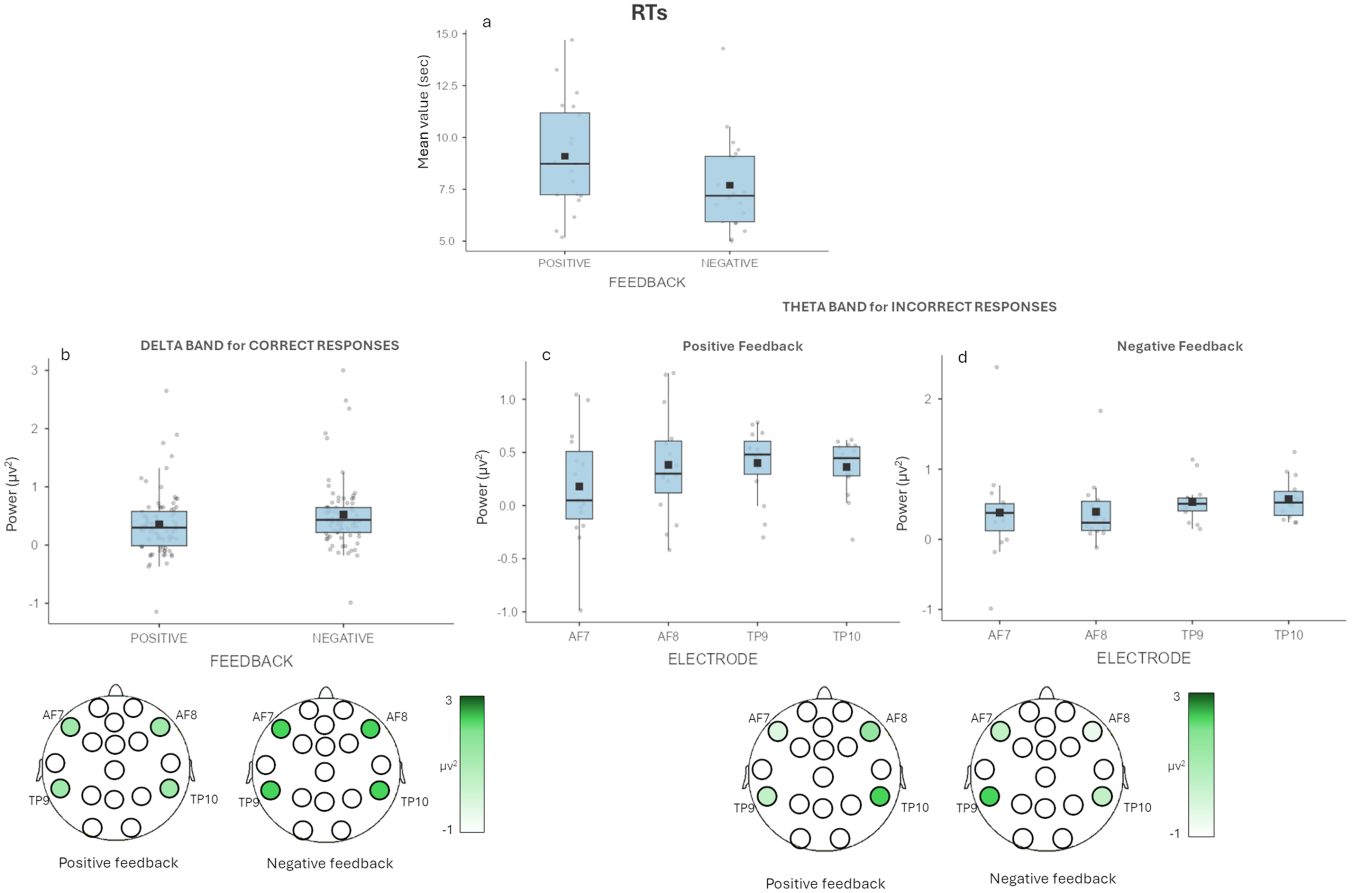
Behavioral results and EEG results for delta and theta. **(a)** The graph shows significant differences for RTs with higher RTs in the recall of the decisions with positive feedback compared to those with negative feedback. **(b)** The graph shows significant differences for correctly recalled decisions for delta band in Feedback. The figure under the graph is a graphic representation of the differences in delta activity during the recall task for positive and negative feedback decisions. **(c-d)** The graph shows non-significant differences in multiple comparison correction for wrongly recalled decisions for theta band in Feedback × Electrode. For each graph bars represent ±1 SE and dots represent observed score.

A main effect was found for Feedback [*F*(1,19) = 7.96, *p* = 0.011, *η*^2^ = 0.079], for which we observed higher RTs for the correctly recalled decisions with a positive (*M* = 9.09, SE = 0.583) compared to negative feedback (*M* = 7.69, SE = 0.506) (Figure 2A).

No other significant effects were found for the ACC of recalled decision, ERRs and RTs of the errors (all *p* > 0.05).

### 3.2 EEG results

#### 3.2.1 Delta band

A main effect for Feedback was found [*F*(1,19) = 13.225, *p* = 0.002, *η*^2^ = 0.022], with higher mean values of delta band for the correct recall of the decisions that in the first phase (i.e., the decision-making phase) received negative feedback (*M* = 0.523, SE = 0.071) compared to those that received a positive feedback (*M* = 0.354, SE = 0.064) (Figure 2B).

No other significant effects were found (all *p* > 0.05). No significant differences were found for wrongly recalled decisions (all *p* > 0.05).

#### 3.2.2 Theta band

A significant interaction effect Feedback × Electrode was found [*F*(3,33) = 4.818, *p* = 0.007, *η*^2^ = 0.026] for the wrongly recalled decisions. In the post-hoc investigating the interaction effect showed: slightly higher values for AF7 for recalled decisions with negative feedback (*M* = 0.391, SE = 0.231) compared to those with positive feedback (*M* = 0.180, SE = 0.145); for AF8 for recalled decisions with positive feedback (*M* = 0.432, SE = 0.112) compared to those with negative feedback (*M* = 0.296, SE = 0.074); for TP9 for recalled decisions with negative feedback (*M* = 0.541, SE = 0.088) compared to those with positive feedback (*M* = 0.404, SE = 0.078) and for TP10 for recalled decisions with negative feedback (*M* = 0.561, SE = 0.084) compared to those with positive feedback (*M* = 0.410, SE = 0.056), but none of them survived multiple comparison correction (Figures 2C,D).

No other significant effects were found (all *p* > 0.05). No significant differences were found for correctly recalled decisions (all *p* > 0.05).

#### 3.2.3 Alpha band

**Figure 3:**
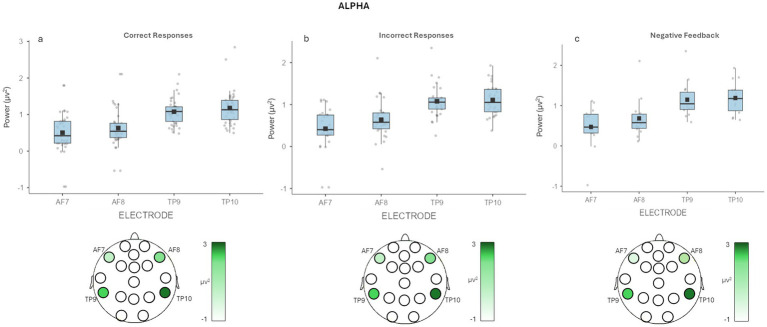
EEG alpha band results. **(a)** The graph shows significant differences for alpha band for correctly recalled decisions in *Electrode*. Bars represent ±1 SE and dots represent observed score. **(b)** The graph shows significant differences for alpha band for wrongly recalled decisions in *Electrode*. The figure under each graph is a graphic representation of the brain area involved during the recall task. Bars represent ±1 SE and dots represent observed score. **(c)** The graph shows significant differences in multiple comparison correction for alpha band in *Feedback* × *Electrode*. Bars represent ±1 standard error and dots represent observed score. The figure under the graph is a graphic representation of the differences in alpha activity during the recall task for negative feedback decisions.

Concerning correctly recalled decisions, a main effect for Electrode [*F*(3, 57) = 9.456, *p* = <0.001, *η*^2^ = 0.268] was detected. Pairwise comparisons revealed higher mean values for alpha band in TP9 (*M* = 1.078, SE = 0.075, *p* = 0.009) and TP10 (*M* = 1.178, SE = 0.103, *p* = 0.007) compared to AF7 (*M* = 1.178, SE = 0.103), and in TP10 compared to AF8 (*M* = 0.630, SE = 0.120, *p* = 0.011) (Figure 3A).

About wrongly recalled decisions, we observed the same main effect for Electrode [*F*(3,33) = 9.99, *p* = <0.001, *η*^2^ = 0.374]. Pairwise comparisons revealed higher values for alpha band in TP9 (*M* = 1.092, SE = 0.104, *p* = 0.035) and TP10 (*M* = 1.143, SE = 0.103, *p* = 0.012) compared to AF7 (*M* = 0.400, SE = 0.160), and in TP10 compared to AF8 (*M* = 0.621, SE = 0.083, *p* = 0.046) (Figure 3B).

Also, it was found a significant interaction effect Feedback × Electrode [*F*(3,33) = 4.86, *p* = 0.007, *η*^2^ = 0.008]. Pairwise comparison showed higher mean values for alpha band in TP10 (*M* = 1.202, SE = 0.125) compared to AF7 (*M* = 0.442, SE = 0.161) for wrongly recalled decisions with negative feedback (*p* = 0.029) (Figure 3C).

No other significant effects were found (all *p* > 0.05).

#### 3.2.4 Beta band

**Figure 4:**
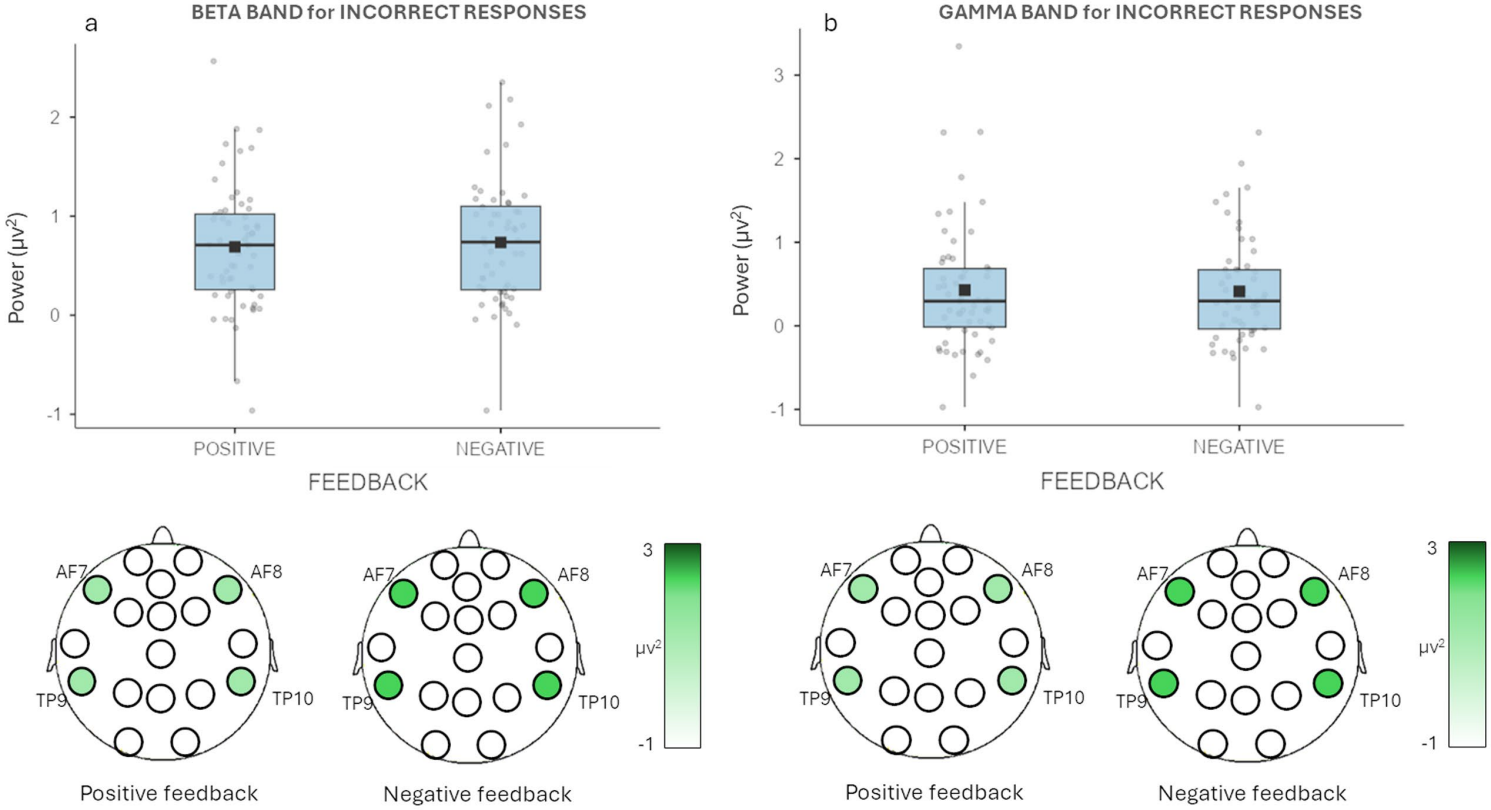
EEG beta and gamma bands results. **(a)** The graph shows significant differences for wrongly recalled decisions for beta band in Feedback. Bars represent ±1 SE and dots represent observed score. **(b)** The graph shows significant differences for wrongly recalled decisions for gamma band in Feedback. Bars represent ±1 SE and dots represent observed score. The figure under each graph is a graphic representation of the differences in beta and gamma activity during the recall task for positive and negative feedback decisions.

For beta band, it was detected a main effect for Feedback [*F*(1,19) = 8.09, *p* = 0.002, *η*^2^ = 0.005], with higher mean values of beta band for wrongly recalled decisions with a negative (*M* = 0.685, SE = 0.087) compared to positive feedback (*M* = 0.608, SE = 0.086) (Figure 4A).

No other significant effects were found (all *p* > 0.05). No significant differences were found for correctly recalled decisions (all *p* > 0.05).

#### 3.2.5 Gamma band

Regarding gamma band, a main effect for Feedback [*F*(1,19) = 7.272, *p* = 0.022, *η*^2^ = 0.003], with higher mean values of gamma band for wrongly recalled decisions with a negative (*M* = 0.362, SE = 0.105) compared to positive feedback (*M* = 0.291, SE = 0.111) (Figure 4A).

No other significant effects were found (all *p* > 0.05). No significant differences were found for correctly recalled decisions (all *p* > 0.05).

## 4 Discussion

The present study examined behavioral – specifically ACC, ERRs and RTs – and EEG data in a sample of decision-makers when recalling prior decisions under two feedback conditions (positive *versus* negative).

Behavioral results only showed higher RTs for accurately recalled decisions with positive feedback compared to those with negative feedback. These results might suggest that attentional biases toward positive information can impact cognitive processing and memory retrieval (Thoern et al., 2016). This influence may result in slower recall times for positive information compared to negative stimuli. Another possible interpretation addressed by García-Arch et al. (2024) is that people tend to prefer receiving feedback that aligns with their self-representation, suggesting that they actively utilize self-consistent information to reinforce self-identity, which may help maintain the stability of self-concept. Nonetheless, we might speculate that decisions with negative feedback are easier to recall since people are more likely to prioritize negative over positive information across various psychological contexts (Norris et al., 2019). Besides, results showed no significant differences in recall accuracy. Indeed, we might speculate that since participants were healthy individuals with typical performance levels, their ACC probably reached a ceiling effect, showing no significant differences across conditions. However, more sensitive measures, including RTs – which are indicators of cognitive effort and decision workload (Balconi et al., 2024a) – and EEG, revealed distinctions between correct and incorrect responses, offering deeper insights into the underlying neural processes.

Regarding EEG results, analysis showed as expected for both correctly and wrongly recalled decisions a general decrease of alpha band mainly in frontal areas (AF7, AF8) compared to temporo-parietal areas (TP9, TP10) in the recall of decisions despite feedback valence. Alpha oscillations indeed function as a gatekeeper in neural processes, playing a crucial role in directing attention and accessing stored information during tasks by managing cognitive load via the inhibition of distractions (Knyazev, 2013). Specifically, suppression of alpha band activity in frontal areas supports top-down control, aiding working memory and attention by filtering distractions and enhancing focus, particularly during recall tasks (Zanto et al., 2011; Payne et al., 2013). In contrast, temporo-parietal alpha activity is associated with memory encoding and maintenance, with higher alpha power improving memory performance (Kimura et al., 2016). Interestingly, results showed low level of alpha band in AF7 compared to TP10 for wrongly recalled decisions with negative feedback suggesting that while frontal areas attempt to control and focus attention, right temporoparietal processing [that might be marked by the right-temporoparietal junction – rTPJ – an area associate with attentional process (Corbetta and Shulman, 2002; Carter and Huettel, 2013)] remains insufficient for accurate recall, especially under conditions of negative feedback that threaten self-concept and may be processed more superficially. Indeed, this pattern of activation could reflect an interaction between the need for self-concept stability and the selective disengagement of attention from self-incongruent feedback, limiting the effective integration of such information (García-Arch et al., 2023).

Conversely, results showed higher delta band activity for correctly recalled decisions with negative compared to positive feedback. Notably, this result is consistent with findings that associate delta oscillations with aversive states, thus episodes linked to positive emotions showed a more pronounced decrease in delta power, while those linked to negative emotions displayed an increase in delta power (Knyazev et al., 2015). Additionally, it is worth noting that delta-increased activity is generalized to the whole scalp and not localized to a specific brain area, which underlines the role of this EEG band in processing and recalling negative stimuli (Balconi et al., 2009; Knyazev et al., 2015; Allegretta et al., 2024).

Regarding wrongly recalled decisions, results showed the implication of high frequency bands beta and gamma. Indeed, analysis showed an increase of beta and gamma for wrongly recalled decisions with negative feedback compared to positive ones. Notably, beta plays a crucial role in supporting top-down control processes in retrieving information from memory (Miller et al., 2018). Additionally, findings show that a decrease in beta power is associated with successful memory formation, particularly in the left inferior prefrontal cortex (Hanslmayr et al., 2011). The modulation of beta oscillations during memory tasks seems connected to encoding and recall processes, with beta wave desynchronization correlating with improved memory performance (Hanslmayr et al., 2014). Furthermore, research shows that beta band activity reflects the cognitive load linked to recalling negative memories, with higher beta power associated with more effortful retrieval processes (Shin et al., 2017). This might indicates that increased beta activity may serve as a neural marker for the cognitive effort needed to access negative memories or alternatively – as shown by findings from ERP studies – where beta-related top-down control and P300 amplitude modulation are tied to valence and expectancy effects (Paul et al., 2022), suggesting that the increased beta activity observed here could signify heightened cognitive effort to process the recall of unexpected negative feedback incongruent with one’s self-representation. Furthermore, similarly to delta, beta did not show significant increase over a specific brain area: indeed, as found by Crespo-García et al. (2022) upon detecting unwanted memories, not a specific area, but several brain areas are engaged (including the anterior-cingulate cortex and the hippocampus) through specific neural oscillatory patterns, thus beta and theta bands.

Notably, it is worth mentioning that theta showed a significant feedback-dependent modulation of electrodes’ activity for the wrongly recalled decisions, showing a generalized higher activity for decisions with negative feedback compared to those with positive feedback in AF7, TP9 and TP10, that however did not survive multiple comparison correction. As highlighted by Crespo-García et al. (2022) theta oscillations in the dorsal anterior cingulate cortex play a crucial role in activating inhibitory control by the right dorsolateral prefrontal cortex during motivated forgetting. Thus, in the case of this study, it is possible that theta signals memory suppression indicating greater control demands for cognitive conflict resolution (Sauseng et al., 2019). However, this speculation might be better addressed by using a high-density EEG in future research.

On the other hand, gamma is associated with encoding and retrieval of specific memory traces, helping the improvement of the accuracy of recalled information (Lundqvist et al., 2016). Research suggests that beta-gamma activity also acts as a “motivational value signal” (HajiHosseini et al., 2012) and increases with heightened attentional resources toward significant events driving behavioral adjustments when feedback is relevant (Li et al., 2016). Therefore, we might speculate that the increased activity of both beta and gamma when wrongly recalling decisions with negative feedback might be interpreted as a neural marker indicating a motivational signal that is perceived as a treat to the self and that therefore prompts a selective forgetting of that self-threatening negative feedback. This speculation might be supported by the MNE (Sedikides and Green, 2005, 2006) which posits that individuals are motivated to protect and enhance their positive self-concept, causing them to process negative, self-threatening feedback less thoroughly. This shallow processing creates fewer memory retrieval pathways, leading to weaker recall of decisions with such feedback. Nonetheless, this selective forgetting might underline a certain level of awareness of the subject in avoiding the recall of a negative event, perceived as self-threatening. Interestingly, as pointed out by Balconi et al. (2023) self-awareness is associated with heightened activity in both the gamma and beta bands, suggesting a synergistic relationship between these oscillations in supporting this process.

Nonetheless, recent studies (García-Arch et al., 2023, 2024) might offer an alternative explanation to the findings of the current study: notably, the differing behavioral and neural responses triggered by recalling a decision that received positive or negative feedback may also be attributed to how well the feedback aligns with the existing self-concept. As pointed out by the authors, self-concept characteristics support psychological continuity and may protect self-representations from being rapidly altered by self-incongruent information (Nowak et al., 2000; Conway, 2005), therefore identity-discrepant inputs are identified early in the processing stages and classified as “false” information (Abendroth et al., 2022). This suggests that the swift detection of self-incongruent feedback serves to safeguard self-representations from disruption by subjectively inaccurate information.Overall, this study reveals key behavioral and EEG factors in recalling decisions under different feedback conditions. Behaviorally, accurate recalls with positive feedback showed longer RTs, likely due to an attentional bias toward positive information (Thoern et al., 2016). Regarding electrophysiological data, reduced alpha activity in frontal areas during both correct and incorrect recalls supports the role of alpha oscillations in managing cognitive load (Knyazev, 2013). Correct recalls with negative feedback showed increased delta activity, linked to aversive states (Knyazev et al., 2015), while incorrect recalls with negative feedback exhibited higher beta and gamma activity, suggesting cognitive effort in processing self-threatening feedback (Miller et al., 2018). This study highlights how feedback valence shapes recall accuracy, RTs, and neural oscillations. However, it is worth mentioning that the positivity bias might be one of different interpretations of the current results. Indeed, as pointed out by García-Arch et al. (2024) in a recent study, there is in healthy adults a behavioral tendency to prioritize the assimilation of feedback that aligns with one’s self-perceptions over feedback that contradicts them. This aligns with the idea that self-beliefs are deeply integrated within a complex network of autobiographical memories, which require stabilizing mechanisms to maintain self-representations and resist contradictory information (Nowak et al., 2000; Conway, 2005). From this perspective, the need to maintain self-concept stability and coherence acts as a strong constraint, influencing which external information is processed and retained, with a preference for information perceived as self-congruent (García-Arch et al., 2023). Therefore, the role of valence may be more complex and potentially secondary to other factors, such as self-congruence, particularly in healthy adult populations. This suggests that the alignment of feedback with self-perceptions could exert a stronger influence on behavioral and neural responses than the mere positive or negative nature of the feedback itself (García-Arch et al., 2024).

While this study is innovative in its multi-method approach to examining the recall of previously made decisions based on feedback valence by integrating behavioral and EEG data, it has some limitations. The primary limitation is its small sample size, which may affect the statistical power of the findings. Future research with a larger participant pool is recommended to validate these results. Moreover, future studies with larger sample size should consider the application of advanced EEG analyses approach using data-driven tests, such as cluster-based permutation test. Secondly, using a wearable EEG device presents limitations: indeed, even if it offers ecological validity, easy setup, and greater comfort with dry electrodes (Ratti et al., 2017; Acabchuk et al., 2021), they also have drawbacks, such as limited scalp coverage, which restricts the examination of broader neural networks (Cannard et al., 2021; Zhang and Cui, 2022; Cannard et al., 2021; Zhang and Cui, 2022). Future studies could improve accuracy and reduce artefacts by using high-density EEG, allowing for more comprehensive mapping of cortical activity related to these processes. Furthermore, future studies could delve deeper into these findings, by incorporating psychometric data through self-report measures examining how individual factors, such as personality traits, decision-making style and maximization tendencies relate to variations in behavioral performance and EEG activity. Another important limitation of the present study is the omission of EEG data collected during the feedback phase. Indeed, these neurophysiological data would have allowed us to associate the brain activity and behavioral patterns observed during recall to processes occurring in the feedback phase, therefore providing a more comprehensive understanding of the mechanisms underlying recall. By focusing solely on the outcomes of the recall phase, our findings are limited to describing the results relative to the recall phase rather than the dynamic processes leading to them. Future studies should consider integrating feedback-related data to enable a more comprehensive analysis of recall and its associated neural and behavioral patterns. Finally, to gain a comprehensive understanding of the implicit components of decision-making, a neuroscientific approach that combines behavioral data with EEG and autonomic measures would be valuable. This approach could clarify the neurophysiological and autonomic responses involved in the recall process, offering insights into cognitive effort and emotional engagement. Moreover, comparing neurophysiological and autonomic responses between the decision-making and recall phases could reveal key differences in processing across these stages.

## Data Availability

The datasets presented in this article are not readily available because the data presented in this study are available on request from the corresponding author due to ethical reasons for sensitive personal data protection (requests will be evaluated according to the GDPR—Reg. UE 2016/679 and its ethical guidelines). Requests to access the datasets should be directed to robertaantonia.allegretta1@unicatt.it.
